# Pseudo-autologous Stem Cell Transplant for the Treatment of Secondary Central Nervous System Lymphoma

**DOI:** 10.7759/cureus.106893

**Published:** 2026-04-12

**Authors:** Dennis L Cooper, Andrew Wollowitz, Jhannine A Verceles, Amanda Lombardo, Mendel Goldfinger, Ioannis Mantzaris, Amit Verma

**Affiliations:** 1 Hematology/Oncology, Montefiore Einstein, Bronx, USA; 2 Emergency Medicine, Montefiore Einstein, Bronx, USA; 3 Medical Oncology, Montefiore Einstein, Bronx, USA

**Keywords:** allogeneic stem cell transplant (allo-sct), autologous stem cell transplant (asct), car t cell therapy, diffuse large b cell lymphoma (dlbcl), pseudo-autologous stem cell transplant (pasct), secondary central nervous system lymphoma

## Abstract

In patients who have previously undergone allogeneic stem cell transplantation and later have stem cells collected for an autologous transplant, the collected cells are sometimes referred to as “pseudo-autologous” to indicate that they are donor-derived. The use of pseudo-autologous stem cell transplantation (pASCT) has rarely been reported.

We describe a case of pASCT in a 66-year-old man with multiple relapses of diffuse large B-cell lymphoma (DLBCL) despite prior chimeric antigen receptor T-cell (CAR T) therapy and a subsequent matched unrelated donor transplant. His most recent relapse after allogeneic transplantation was limited to the central nervous system (CNS) and presented with multiple cranial nerve palsies. Although previously collected autologous stem cells and donor-derived allogeneic stem cells were available, pseudo-autologous stem cells were collected during recovery from a modified salvage chemotherapy regimen (methotrexate (MTX), cytarabine, thiotepa, and rituximab (MATRix)). He subsequently received high-dose therapy with busulfan and thiotepa followed by pASCT. Despite receiving only brief (<1 month) graft-versus-host disease (GVHD) prophylaxis, he did not develop GVHD and remains disease-free 23 months after transplant.

We discuss the rationale for selecting pASCT despite the availability of stored autologous and allogeneic stem cells.

## Introduction

Although 60%-70% of patients with diffuse large B-cell lymphoma (DLBCL) are cured with frontline therapy, 30%-40% either have a poor response or experience relapse. Recent studies have shown that chimeric antigen receptor T-cell (CAR T) therapy is superior to high-dose chemotherapy and autologous transplant in patients with primary refractory disease or relapse within one year of treatment [1,2].

In patients who achieve complete remission (CR) for ≥1 year, high-dose therapy followed by autologous stem cell transplant (ASCT) remains the preferred approach, with subsequent relapses treated with CAR T therapy in eligible patients. The management of patients who relapse after CAR T therapy has increasingly shifted toward immune-based therapies rather than conventional chemotherapy. Examples include bispecific antibodies, engineered molecules with two binding sites that simultaneously target a tumor antigen and a T-cell receptor to promote cytotoxic activity [3], and antibody-drug conjugates, in which a monoclonal antibody delivers a cytotoxic payload to tumor cells (e.g., loncastuximab tesirine) [4].

Allogeneic stem cell transplantation may be offered to medically fit patients who achieve a response or stable disease [5]. For patients who relapse after both CAR T therapy and allogeneic transplant, treatment options are limited and are often primarily supportive.

In this report, we describe the first case of pseudo-autologous stem cell transplantation (pASCT) in a patient with recurrent DLBCL involving the central nervous system (CNS) after multiple lines of therapy, including CAR T therapy and allogeneic transplant.

## Case presentation

Initial presentation

A 57-year-old male physician presented with a two-month history of a 5 cm subcutaneous mass over the right hip. Biopsy demonstrated high-grade DLBCL, germinal center subtype, with a Ki-67 proliferative index of 90%-95%. Initial staging with positron emission tomography-computed tomography (PET-CT) and bone marrow biopsy showed no additional sites of disease involvement. He received six cycles of rituximab, cyclophosphamide, doxorubicin, vincristine, and prednisone (R-CHOP), achieving CR.

First relapse

**Figure 1:**
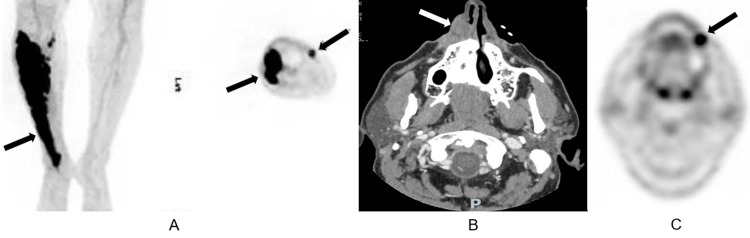
Recurrences of DLBCL (A) PET-CT scan, pre-CAR T cell therapy, showing FDG avidity (high glucose metabolism) of a large muscular area of the right leg (coronal plane left, transverse plane right). (B) CT scan of facial bones showing a large soft tissue mass along the right nose and maxillary bone. (C) PET-CT scan showing FDG avidity of the left buccal area. DLBCL: diffuse large B-cell lymphoma, PET-CT: positron emission tomography-computed tomography, CAR T: chimeric antigen receptor T-cell, FDG: fluorodeoxyglucose.

Three years after completing chemotherapy, he developed severe pain and swelling in the right tibial region. A PET-CT scan (Figure 1A) was suspicious for recurrent disease, which was confirmed by biopsy. He received two cycles of salvage rituximab, ifosfamide, carboplatin, and etoposide (R-ICE) in preparation for ASCT. However, during the interval between stem cell mobilization and the planned initiation of high-dose therapy, he developed recurrent disease in the anterolateral right leg. Treatment was then changed to CAR T therapy with axicabtagene ciloleucel, resulting in a CR.

Second relapse

Twenty-nine months after CAR T therapy, CT imaging of the facial bones revealed a soft tissue mass at the right nasal alae extending to the frontal process of the right maxilla (Figure 1B). A biopsy revealed recurrence. PET-CT was positive only for the facial lesion and a few small dermal nodules at the inner right calf. He received radiation therapy (RT) and enrolled in a phase 1a/1b clinical trial with rituximab plus a novel CD47-blocking agent, TTI-622-01 (CD47 is over-expressed on tumor cells and suppresses phagocytosis by macrophages). 

Third relapse

After six cycles of clinical trial therapy, he developed a trigeminal neuralgia-like syndrome, followed by the rapid appearance of a left buccal mass, which was confirmed to be DLBCL (Figure 1C). Following additional RT, he received three cycles of salvage chemotherapy with polatuzumab, bendamustine, and rituximab [6]. This was followed by a reduced-intensity, matched unrelated donor allogeneic stem cell transplant using fludarabine, melphalan, and 2 Gy total body irradiation as conditioning.

Fourth relapse

Approximately eight months after transplant, he developed decreased hearing and tinnitus in the left ear, associated with loss of balance, consistent with an eighth cranial (vestibulocochlear) nerve lesion. Magnetic resonance imaging (MRI) demonstrated abnormal enhancement in the left internal auditory canal (IAC), which was initially suspected to be of viral etiology and was treated with corticosteroids. However, his symptoms rapidly progressed to include hearing loss in the right ear, and a repeat MRI demonstrated bilateral enhancement of the IACs. Given high suspicion for leptomeningeal lymphoma, he was treated emergently with base-of-skull RT, which preserved right-sided hearing but resulted in no improvement on the left. Lumbar puncture revealed mildly elevated protein but no abnormal cells on cytology, with insufficient cells for flow cytometry. PET-CT showed no evidence of systemic disease.

He was subsequently treated with high-dose intravenous methotrexate (MTX), which was complicated by a brief episode of acute kidney injury. 

Further CNS disease progression

Despite additional treatment with intermediate-dose MTX, two months later, he developed sudden-onset diplopia and facial droop, consistent with left sixth (abducens) and right seventh (facial) cranial nerve palsies. He was emergently hospitalized and treated with three doses of intrathecal (IT) MTX and hydrocortisone, followed by salvage chemotherapy with MTX, cytarabine (Ara-C), thiotepa, and rituximab (MATRix) [7], resulting in CR of the cranial nerve deficits. As he recovered from a neutropenic nadir, pseudo-autologous stem cells were collected. These were confirmed to be donor-derived, as repeat chimerism studies demonstrated 100% donor hematopoiesis. He subsequently received two additional cycles of intermediate-dose MTX along with further IT chemotherapy. Cerebrospinal fluid analysis was negative except for mildly elevated protein. Repeat MRI showed improvement in the previously noted internal auditory canal abnormalities, and PET-CT showed no evidence of disease elsewhere.

High-dose chemotherapy and pASCT

The patient received high-dose therapy with busulfan 3.2 mg/kg (days -7 and -6) and thiotepa 5 mg/kg (days -5 and -4), followed by pASCT. Pharmacokinetic adjustment of busulfan was not performed. This regimen has been shown to be tolerable and effective in patients older than 65 years with primary central nervous system lymphoma [8]. He received single-agent sirolimus briefly for graft-versus-host disease (GVHD) prophylaxis, achieved neutrophil engraftment on day 15, and was discharged on day 18. Aside from a one-day hospitalization for bilateral inguinal hernia repair, he has not required readmission. He is now more than 23 months post-pASCT, remains employed full-time, and has no evidence of disease recurrence or GVHD. In addition to improvement in balance and dysacusis (qualitatively abnormal hearing), he has resumed bicycling and skiing.

## Discussion

For CNS recurrence of DLBCL, high-dose chemotherapy followed by ASCT [9] and CAR T therapy [10] are the only potentially curative treatment options. However, because the disease recurred after CAR T therapy, this approach was not reconsidered. In contrast, although the patient was heavily pretreated, he had not previously received high-dose therapy with agents that cross the blood-brain barrier. For marrow rescue after high-dose therapy, several stem cell sources were available, including previously stored autologous (host-derived) stem cells, donor-derived allogeneic stem cells, and the pseudo-autologous cells ultimately used. True autologous stem cells have occasionally been used after allogeneic transplant for graft failure or treatment-refractory GVHD, but with limited success [11]. For example, the European Group for Blood and Marrow Transplantation (EBMT) evaluated 35 patients who received autologous backup grafts (25 for graft failure, 8 for refractory GVHD, and 2 for relapse). One-year survival was 16% for graft failure and 13% for GVHD. In addition, because this patient had an extranodal relapse around the time of stem cell collection, tumor contamination of a true autologous product would be a significant concern. In one study, 50% of patients with DLBCL in remission had detectable lymphoma cells in the mobilized stem cell product [12].

With respect to the decision not to use the stored donor (allogeneic) product, infusion of an unselected allogeneic graft would carry a substantial risk of GVHD despite prophylaxis, as the CD3+ T-cell content in a stem cell graft is 1-2 logs higher than that used in even the most intensive donor lymphocyte infusions [13]. In this case, there was no evidence of GVHD despite only brief prophylaxis with single-agent sirolimus. The absence of GVHD following pseudo-autologous stem cell transplantation has been described in four prior cases [14-17] and is thought to reflect immune tolerance (Table 1). Specifically, donor-derived T cells collected long after allogeneic transplantation may become “acclimated” or tolerized to the host, reducing the likelihood of GVHD despite their donor origin. 

**Table 1:** Prior cases of pseudo-autologous transplant GVHD: graft-versus-host disease.

Reference	Disease treated	GVHD	Outcome
Hatsuse et al. [14]	Recurrent multiple myeloma after allogeneic transplant	No prophylaxis; no GVHD	Partial response
Palfreyman et al. [16]	Recurrent multiple myeloma after allogeneic transplant	No prophylaxis; no GVHD	Partial response
Tamaki et al. [17]	Donor-derived mantle cell lymphoma after allogeneic transplant	No prophylaxis; no GVHD	Complete response >1 year
Jaing et al. [15]	Inoperable brain tumor after prior umbilical cord blood transplant	No prophylaxis; no GVHD	Complete remission 3 years

## Conclusions

We believe this is the first report of the use of pASCT in a patient with relapsed DLBCL, and it supports the safe and effective use of pASCT in selected patients who may benefit from high-dose therapy after prior allogeneic stem cell transplantation.
